# Antimalarial Activity and Mechanisms of Action of Two Novel 4-Aminoquinolines against Chloroquine-Resistant Parasites

**DOI:** 10.1371/journal.pone.0037259

**Published:** 2012-05-23

**Authors:** Anna Caroline Campos Aguiar, Raquel de Meneses Santos, Flávio Júnior Barbosa Figueiredo, Wilian Augusto Cortopassi, André Silva Pimentel, Tanos Celmar Costa França, Mario Roberto Meneghetti, Antoniana Ursine Krettli

**Affiliations:** 1 Centro de Pesquisas René Rachou, Fundação Oswaldo Cruz, Belo Horizonte, Minas Gerais, Brazil; 2 Programa de Pós Graduação em Medicina Molecular, Faculdade de Medicina, Universidade Federal de Minas Gerais, Belo Horizonte, Minas Gerais, Brazil; 3 Instituto de Química e Biotecnologia, Universidade Federal do Alagoas, Maceió, Alagoas, Brazil; 4 Laboratório de Modelagem Molecular Aplicada a Defesa Química e Biológica (LMDQB), Instituto Militar de Engenharia, Rio de Janeiro, Rio de Janeiro, Brazil; 5 Departamento de Química, Pontifícia Universidade Católica do Rio de Janeiro, Rio de Janeiro, Rio de Janeiro, Brazil; Museum National d'Histoire Naturelle, France

## Abstract

Chloroquine (CQ) is a cost effective antimalarial drug with a relatively good safety profile (or therapeutic index). However, CQ is no longer used alone to treat patients with *Plasmodium falciparum* due to the emergence and spread of CQ-resistant strains, also reported for *P. vivax*. Despite CQ resistance, novel drug candidates based on the structure of CQ continue to be considered, as in the present work. One CQ analog was synthesized as monoquinoline (MAQ) and compared with a previously synthesized bisquinoline (BAQ), both tested against *P. falciparum in vitro* and against *P. berghei* in mice, then evaluated *in vitro* for their cytotoxicity and ability to inhibit hemozoin formation. Their interactions with residues present in the NADH binding site of *P falciparum* lactate dehydrogenase were evaluated using docking analysis software. Both compounds were active in the nanomolar range evaluated through the HRPII and hypoxanthine tests. MAQ and BAQ derivatives were not toxic, and both compounds significantly inhibited hemozoin formation, in a dose-dependent manner. MAQ had a higher selectivity index than BAQ and both compounds were weak *Pf*LDH inhibitors, a result previously reported also for CQ. Taken together, the two CQ analogues represent promising molecules which seem to act in a crucial point for the parasite, inhibiting hemozoin formation.

## Introduction

Malaria is one of the most prevalent parasitic diseases in the world. It affects approximately 500 million individuals throughout the tropical and subtropical areas of developing countries and causes considerable morbidity and mortality with about 800,000 deaths worldwide each year [1]. *P. falciparum* infection in non-immune adults and children is often associated with severe cerebral malaria. The global importance of this disease, current limitations of vector control and the absence of an effective vaccine, makes the development of therapeutic antimalarial drugs the main strategy of malaria control [2].

CQ is a cost effective antimalarial drug with a relatively good safety profile [3]. However, CQ is no longer used alone due to the emergence and spread of *P. falciparum* CQ-resistant strains and, more recently, of *P. vivax* [4]–[6]. Artemisinin-based combination therapy (ACT) is the first line treatment in *P. falciparum* malaria (WHO, 2009). However, the limited availability of ACT and the decreased susceptibility of *P. falciparum* to artemisinin derivatives [7]–[8] have required the development of novel antimalarial drugs [9]–[11]. Previous studies have described the discovery of novel antimalarial drugs through analysis of medicinal plants [12] and through novel drug synthesis protocols [13]–[14], however, no new active compound has been shown to be as effective as CQ.

Despite the resistance of *P. falciparum* to CQ, novel drug candidates based on the structure of CQ continue to be considered [15]–[18]. In the present work, CQ analogs were synthesized as mono- and bisquinoline-based derivatives, referred as MAQ and BAQ, respectively. The main structural aspects considered included the maintenance of the 4-aminoquinoline pharmacophore group and the presence of proton-accepting sites to increase drug bioavailability in the digestive vacuole of the parasite. The compounds were tested: (i) as blood schizonticides against *P. falciparum in vitro* and against *P. berghei* malaria in mice; (ii) for their *in vitro* cytotoxicity; (iii) for their ability to inhibit hemozoin formation; and (iv) for their binding mode to lactate dehydrogenase and dimeric hematin *in silico*.

## Results

### Synthesis and characterization of CQ analogs

The structure of the two aminoquinoline derivatives, MAQ and BAQ, and their synthesis strategy are depicted in Scheme S1. Both CQ analogs possess the conserved 4-aminoquinoline moiety (a crucial pharmacophore group), and have a chlorine substituent at the 7 position on the quinoline ring, and, both have amino groups on the alkyl chain [19]. MAQ, a new monoquinoline molecule, and BAQ, a previously synthesized bisquinoline already tested against *P. falciparum* and proven to be active [20], were prepared from 4,7-dichloroquinoline and diethylenetriamine. These reactions occurred via a S_N_Ar synthesis step, which eliminated the use of solvents [21]. MAQ and BAQ were obtained by controlling the stoichiometric relationship between these reagents. The synthesis protocol for the bisquinoline compound BAQ was described previously [20].

**Table 1:** The anti-*P. falciparum* activities of BAQ and MAQ determined in parallel with chloroquine, by the ELISA anti-HRPII assay or by ^3^H hypoxanthine incorporation.

Compounds	W2 (CQ resistant)		3D7 (CQ sensitive)	
IC_50_ (nM)	HRPII	Hypoxanthine	HRPII	Hypoxanthine
BAQ	550±0.0	60±20	35±8	105±7
MAQ	260±20	320±70	26±2	66±5
CQ	120±0.0	180±0.0	6.9±0.9	7.4±1.2

All compounds were active at nanomolar doses in both tests.

IC_50_ = dose that inhibits 50% of blood parasites growth, evaluated in three or four different experiments for each test.

BAQ and MAQ were isolated as white solids, which underwent a satisfactory elemental analysis and were fully characterized by NMR and IR spectroscopy. On the ^1^H NMR spectrum, MAQ showed five of the expected signals of the aromatic region (between 8.32 and 6.50 ppm) and four signals related to the methylenic groups (between 3.47 and 2.78 ppm). The hydrogen signals of the amino groups were either not present or had an integration level lower than the predicted value due to the fast H/D exchange with the deuterated solvent. In the ^13^C NMR spectrum, it was possible to identify nine signals related to the aromatic carbons (between 152 and 97 ppm) and the four signals associated with the methylenic carbons (50 to 40 ppm). In the infrared spectrum of MAQ, the typical absorption bands for this kind of chemical structure were observed and the ^1^H NMR spectrum was consistent with the five typical aromatic signals (between 8.25 and 6.46 ppm). However, only two signals related to the methylenic groups were observed due to the symmetry of the molecule (3.45 and 2.95 ppm). In the ^13^C NMR spectrum of BAQ, the expected nine signals related to the aromatic carbons (between 152 and 97 ppm) and the two possible signals of the methylenic carbons (46.8 and 41.8 ppm) were observed. The infrared spectrum of BAQ exhibited the absorption bands predicted for this structure.

### BAQ and MAQ activities against *P. falciparum in vitro*

The 4-aminoquinoline derivatives MAQ and BAQ were compared with CQ for their antiplasmodial activities against the W2 clone of *P. falciparum* (CQ-resistant and mefloquine-sensitive) and against a CQ-sensitive strain 3D7. All compounds showed activity in the nanomolar range in the HRPII and hypoxanthine tests (Table 1). The IC_50_ values were similar in both assays, although somewhat lower for BAQ in the hypoxanthine test with W2 parasites. As expected, the IC_50_ values of BAQ and MAQ were lower with the CQ- sensitive 3D7 strain than with W2 CQ- resistant in most tests.

Neither MAQ or BAQ were toxic against hepatoma (HepG2) or kidney (BGM) cell lines, and important, the new CQ analog MAQ was less toxic than BAQ, based on MTT assay (Table 2) or neutral red method (data not shown). Additionally, the CQ analogs were not lytic to human erythrocytes. Even at doses 100 times higher than the IC_50_ values no hemolysis was detected.

**Table 2:** Cytotoxicity of BAQ and MAQ against a human hepatoma cell line (HEPG2) and a monkey kidney cell line (BGM) determined by the MTT assay.

Compounds	MDL_50_ (µM)		Selectivity index*	
	BGM	HepG2	BGM	HepG2
BAQ	50±30	50±0.0	90	90
MAQ	100±0.0	270±10	384	1038
CQ	370±0.0	490±0.0	3083	4083

Data expressed as the minimal lethal dose for 50% of cells (MDL50), used to calculate the selectivity index against either cell.

* Selectivity index = MDL_50/_/IC_50_.

The selectivity index- IS (the ratio between MDL_50_ and IC_50_) was determined and both compounds had an IS greater than 90. The IS was particularly high for MAQ, 384 and 1038, against BGM and HepG2 cells respectively (Table 2). Nevertheless, neither compound was better then CQ (IS>3083)

### BAQ and MAQ were also active *in vivo* against rodent malaria

The antimalarial activity of the new CQ analogs was evaluated in mice with *P. berghei* and is summarized in Table 3. MAQ and BAQ were active *in vivo* at a dose of 50 mg/kg on days 5, 8 and 10 days post-infection, at 25 mg/kg only MAQ was active. MAQ caused a 95% reduction of parasitemia on day 5. However, only CQ caused 100% suppression of parasitemia and increased the animal survival, but the survival differences were not significant from the other groups.

**Table 3:** Antimalarial activity of BAQ and MAQ in mice infected with *P. berghei* after treatment with daily doses of the compounds during three consecutive days.

Compounds	Dose (mg/kg)	Percent reduction of parasitemia*			Time of survival (in days)
		5°	8°	10°	
BAQ	25	64	26	37	25±2
	50	81	75	43	24±1
MAQ	25	91	61	70	24±1
	50	95	71	76	25±1
CQ	20	100	100	100	>30
Control	0	0	0	0	19±7

* Reductions ≤30% were considered as inactive, 30–50% as partially active and ≥50% as active drugs.

### The antimalarial activity of BAQ and MAQ involves inhibition of hemozoin formation

**Figure 1:**
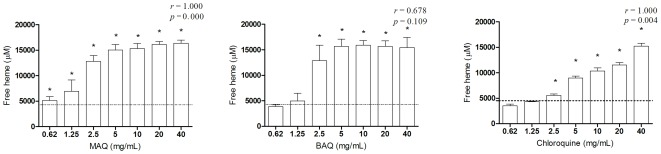
Inhibition of hemozoin formation by the CQ analogs BAQ and MAQ (mean ± SD from triplicates), in two different experiments. Statistical differences as compared to drug-free controls are indicated in each graph by an asterisk (p<0.05). The p and r values represent the statistical correlation analyses.

Whether anti *P. falciparum* activity of BAQ and MAQ involves inhibition of hemozoin formation was evaluated using an indirect *in vitro* test. Both compounds significantly inhibited hemozoin formation *in vitro* (Figure 1) in a dose-dependent reaction. The inhibition was more evident with MAQ than with BAQ or CQ (Figure 1A).

**Figure 2:**
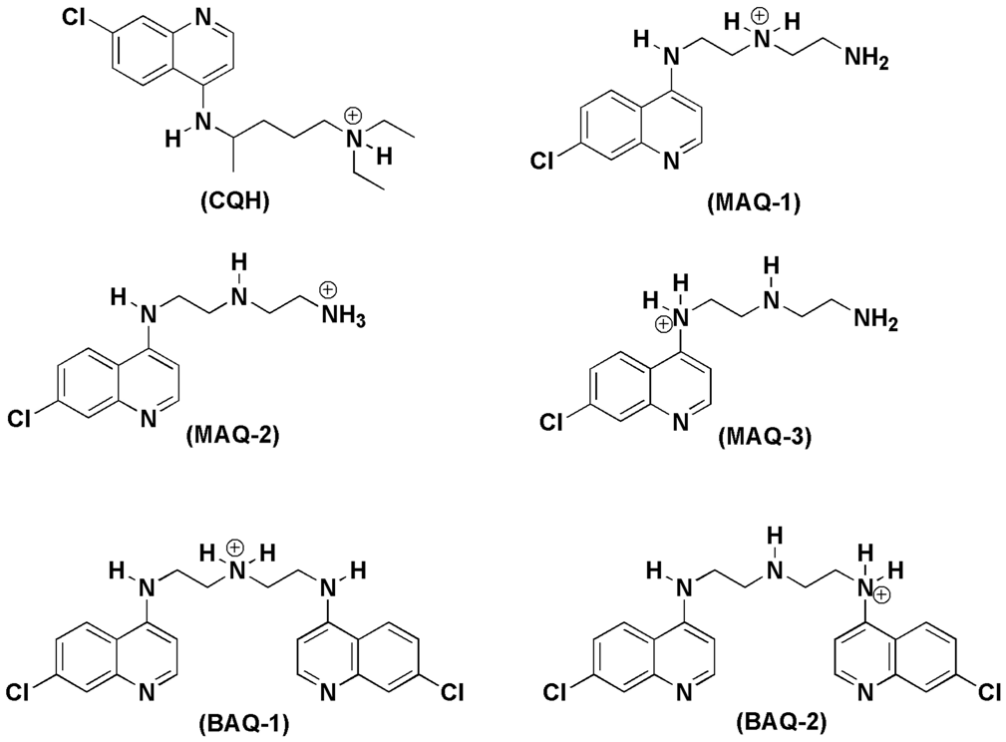
Chemical structures of the protonated forms of the CQ analogs BAQ and MAQ.

**Figure 3:**
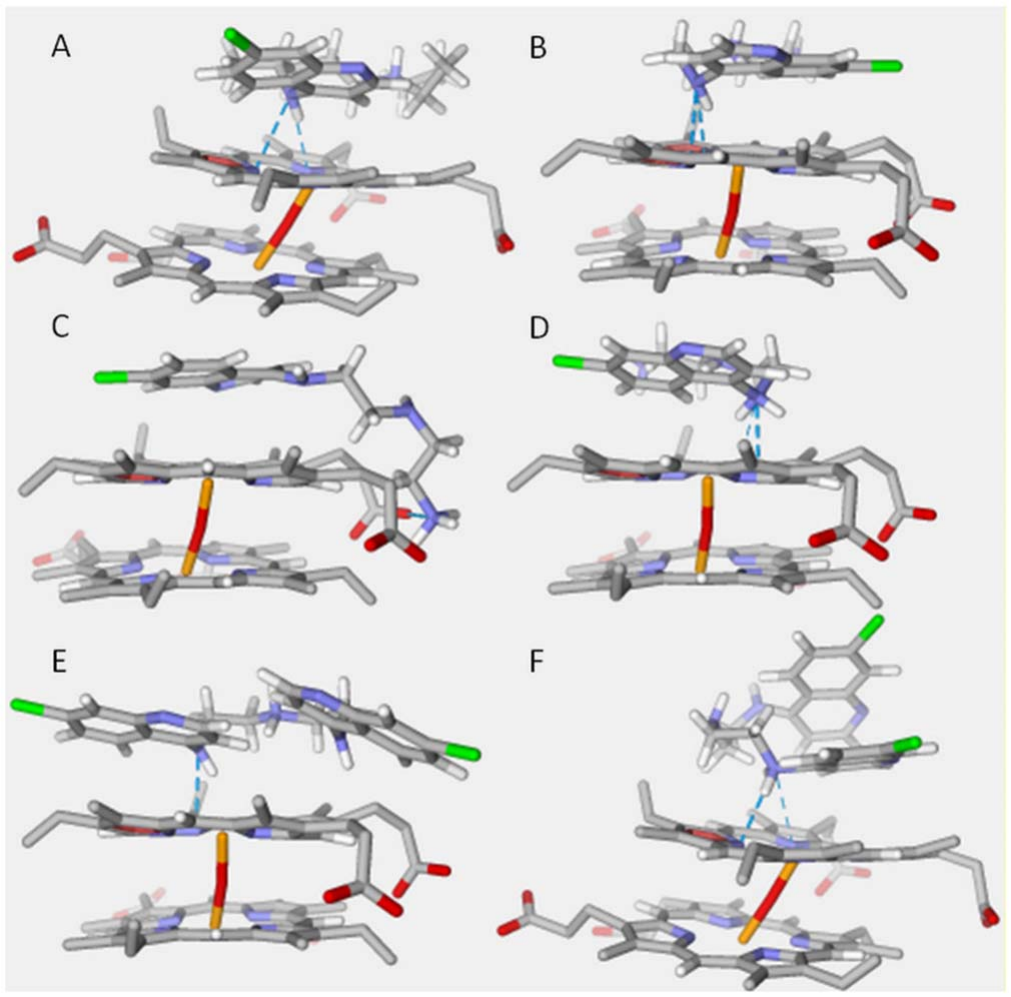
Compounds docked in dimeric hematin. (A) Protonated CQ, (B) MAQ-1, (C) MAQ-2, (D) MAQ-3, (E) BAQ-1, (F) BAQ-2.

Similarly, docking studies with MAQ and BAQ showed that the MolDock Score energies between the antimalarials and the dimeric hematin (Table 4) were approximately –100.000 kcal mol^–1^. Both aminoquinoline derivatives, similar to CQ, were able to interact with dimeric hematin to form a complex. The best conformations (Figure 2) obtained for the complexes showed that the aromatic rings of both compounds were parallel to the hematin ferriprotoporphyrin group (Figure 3). Weak hydrogen bonds (approximately −5.0 kcal mol^–1^) corroborate previous studies demonstrating that the hydrophobic interactions were the main contributors for the binding between CQ and the dimer [22]–[23].

**Table 4:** Docking energies of the protonated forms of the CQ analogs BAQ and MAQ.

Structure	H Bond Energy (kcal mol^−1^)	MolDock Score (kcal mol^−1^)
Protonated CQ	−3.43	−105.97
MAQ-1	−1.95	−100.57
MAQ-2	−5.00	−92.16
MAQ-3	−3.50	−76.50
BAQ-1	−2.50	−119.69
BAQ-2	−2.50	−116.89

### The antimalarial action of BAQ and MAQ involves binding to lactate dehydrogenase

**Figure 4:**
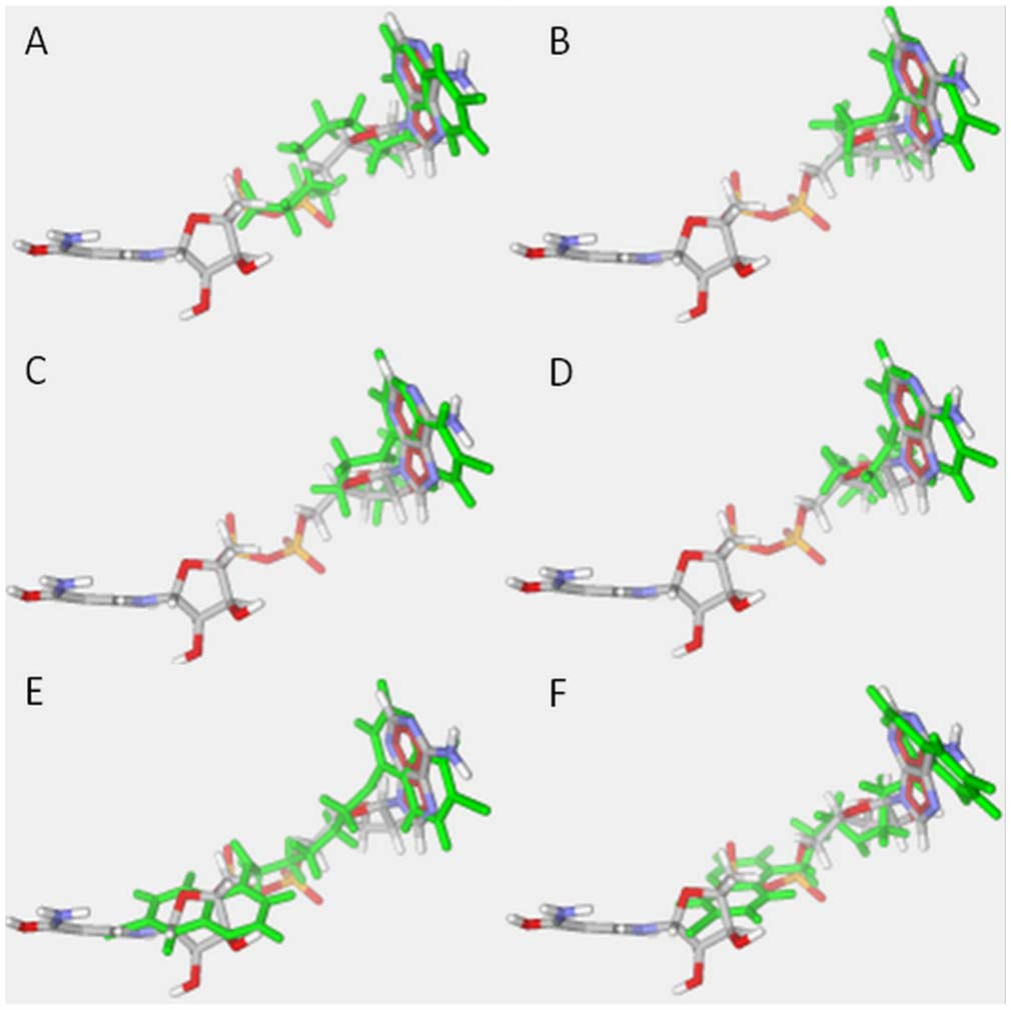
Best conformations of the protonated forms of chloroquine, MAQ and BAQ (in green) in the binding pocket of NADH (in CPK) as generated by the MVD® software. (A) Protonated chloroquine, (B) MAQ-1, (C) MAQ-2, (D) MAQ-3, (E) BAQ-1, (F) BAQ-2.

Table 5 presents the H-bond energy and MolDock Scores of the residues involved in H-bonds with NADH as well as the protonated forms of chloroquine, BAQ and MAQ docked to PfLDH. Figure 4 shows that these compounds presented the most stable energy conformations in the binding site of NADH. This suggests that they are NADH competitors. The H-bond energy between the protonated chloroquine and the enzyme was −3.8 kcal mol^−1^, a high value compared with that of NADH H-bond energy (−35.3 kcal mol^−1^). The energy of interaction between the enzyme and the protonated forms of MAQ were approximately −250.00 kcal mol^−1^. Furthermore, MAQ like the protonated chloroquine, bound to the active site of PfLDH; BAQ-1 and BAQ-2 presented the lowest energies, which were extremely large when compared to NADH. These findings suggest that all these compounds are weak inhibitors of PfLDH, as previously reported for chloroquine [24].

**Table 5:** Docking results of NADH and the protonated forms of chloroquine, BAQ and MAQ in the active site of *Pf*LDH.

Ligands	H Bond Energy (Kcal mol^−1^)	MolDock Score (Kcal mol^−1^)	Residues (H Bond interactions)
NADH	−35.3	−432.3	Asp53, Thr97, Gly29, Tyr85, Leu25, Met30, His126, Val55, Glu122, Phe100, His195
Protonated Chloroquine	−3.8	−252.4	Gly99
MAQ-1	−8	−247.5	Asp53, Gly99
MAQ-2	−2.9	−266.2	Asp53, Gly99
MAQ-3	−0.5	−249	Asp53
BAQ-1	−17.4	−337.2	Gly99, Thr97, Asp53, Asn140
BAQ-2	0	−297.3	-

## Discussion

A large series of bisquinoline derivatives containing proton-accepting amino groups on the aliphatic chain moiety has already been described [19] and considered a promising class of compounds for treating drug-resistant malaria parasites. Two CQ analogs MAQ and BAQ were obtained as low-cost antimalarial prototypes, which is important considering that disease transmission is concentrated in developing countries and is most frequent among the impoverished classes of several endemic countries [9]. Comparisons between their antimalarial activities aimed to evaluate the molecular structural-activity relationship between mono- and bisquinoline compounds.

We showed that MAQ and BAQ are active *in vitro* against CQ-resistant and CQ-sensitive *P. falciparum* parasites at nanomolar doses in the hypoxanthine incorporation test and in an enzyme-linked immunosorbent assay (ELISA), using antibodies against the histidine-rich protein 2 (HRP-II), a parasite protein.Our results show that MAQ, a new monoquinoline, has a higher IS, in consonance with the tendency of a decreased toxicity when comparing mono- to bisquinoline analogs. Such *in vitro* activity was confirmed *in vivo* in mice infected with *P. berghei*; MAQ was more effective as proven by parasitemia reduction in response to drug treatment.

Further *in vitro* analysis showed that the compounds interacted with dimeric hematin and inhibited heme polymerization. Although the mechanism of action of CQ is not well understood, several experimental studies describe the interaction between CQ and the dimeric form of heme [25], [26], [27], [28]. This interaction seems responsible for the inhibition of hemozoin formation, a hypothesis further supported by docking studies, which also suggest that all protonated forms of MAQ and BAQ interact with the rings parallel to the heme group, as described previously for CQ [26], [27], [28], [29].

The presence of two quinoline moieties in BAQ increases the probability of hydrophobic interactions when compared with MAQ and also results in lower docking energies for all protonation states of the amino groups. The results of this study suggest that the protonated BAQ at the central nitrogen (BAQ-1) should be more effective at inhibiting heme polymerization.

Experimental and modeling studies have shown that chloroquine binds to PfLDH, suggesting that CQ possesses another mechanism of drug action or a mechanism complementary to the one already proposed [24], [29]. Although PfLDH is not a direct chloroquine target, experimental data have shown its weak binding to chloroquine [30]. The existence of this shared target-binding site suggests that PfLDH is related to drug effectiveness. The docking data (Figure 4) suggest that the protonated forms of BAQ and MAQ present the most stable energetic conformations at the binding site of NADH inside PfLDH. The H-bond energy and MolDock Scores, the residues involved in the H-bonds with NADH and the protonated forms of chloroquine as well as BAQ and MAQ binding to PfLDH (Table 5) suggest that these compounds are NADH competitors. The H-bond energy between protonated chloroquine and the enzyme is −3.8 kcal mol^−1^, a high value compared with the H-bond energy of NADH (−35.3 kcal mol^−1^). The energies of interaction between the enzyme and the protonated forms of MAQ were approximately –250.0 kcal mol^−1^. Furthermore, MAQ compounds showed a similar behavior at the active site of PfLDH compared with protonated chloroquine. BAQ-1 and BAQ-2 possessed the lowest energies, which were extremely large compared with NADH. Overall, these findings suggest that all of these compounds are weak inhibitors of *Pf*LDH, as previously reported for chloroquine [22]–[23].

One of the recent strategies to develop effective antimalarial agents is based on the development of metal-drugs [31], [32], [33]. Thus, MAQ and BAQ were associated with metals readily available to be tested for their antimalarial activity, in promising ongoing tests (data not shown). Metallocomplexed drugs, considered first-line anticancer drugs [34], [35], are increasingly used to develop anti-parasitic agents [36], [37], [38].

## Materials and Methods

### Reagents and drug synthesis

All reactions for drug synthesis were performed under a 100% argon atmosphere using a dual vacuum/argon line and standard Schlenk techniques. Reagents and solvents were purchased from Sigma Aldrich and used without further purification. The IR spectra were recorded on a Varian 640-IR with an ATR device and the elementary analyses were conducted at the analytical center, University of São Paulo. The ^1^H NMR spectra were recorded at 400.130 MHz and the ^13^C NMR spectra at 100.613 MHz on a Bruker instrument (Bruker Avance 400) and were externally referenced to the tetramethylsilane (TMS). Chemical shifts (δ) and coupling constants (*J*) were expressed in ppm and Hz, respectively. The melting or decomposition points of the structures were determined on a MQAPF 302 apparatus.

### Synthesis of the 4-aminoquinolines, MAQ and BAQ

MAQ, *N^1^-(2-aminoethyl)-N^2^-(7-chloroquinol-4-yl)ethane-1,2-diamine*, was prepared according to the synthesis procedures described by Musonda and collaborators [39]. Briefly, a mixture of 4,7-dichloroquinoline (0.98 g, 4.9 mmol) and diethylenetriamine (2.7 mL, 25 mmol) was heated at 80°C for 1 h without stirring, following by heating at 135°C for 3 h with stirring. The mixture was cooled to room temperature, alkalized with an aqueous solution of 10% NaOH (30 mL), extracted with hot EtOAc (3×50 mL) and dried over Na_2_SO_4_. The solvent was removed under vacuum and the residue was crystallized with CH_2_Cl_2_/hexane, thereby leading to a white solid. Chemical and spectral analyses revealed the following: Yield: 0.52 g, 40%. M.F: C_13_H_17_ClN_4_; M.M.: 264.75 g/mol. Elem. Anal., theoretical: C_13_H_17_ClN_4_, C, 58.98; H, 6.47; N, 21.16, experimental: C_13_H_17_ClN_4_.H_2_O, C, 55.23; H, 6.54; N, 19.22. M.P.: 99–100°C; I.R. (ν_máx_ cm^−1^): 3256 (Ar- NH); 3065 (CH quinoline ring); 2929 and 2839 (CH_as/s_ and CH_2_); 1607, 1577 and 1528 (C = C quinoline ring); 1449 (CH_2_); 1280 (Ar-C-N); 1133 (C-N); 874, 795 and 761 (δ = C-H out-of-plane aromatic ring). RMN ^1^H (400 MHz, CD_3_OD): δ 1.93 (s, NH amine), 2.78 (t, *J* = 5.76 Hz, 2H, CH_2_-16), 2.85 (t, *J* = 5.76 Hz, 2H, CH_2_-15), 2.95 (t, *J* = 6.38 Hz, 2H, CH_2_-13), 3.47 (t, *J* = 6.38 Hz, 2H, CH_2_-12), 3.70 (ls, NH aniline), 6.50 (d, *J* = 5.71 Hz, 1H, Ar-*H* C-3), 7.35 (dd, *J* = 9.01 Hz, *J* = 2.14 Hz, 1H, Ar-*H* C-6), 7.73 (d, *J* = 2.14 Hz, 1H, Ar-*H* C-8), 8.07 (d, *J* = 9.01 Hz, 1H, Ar-*H* C-5), 8.32 (d, *J* = 5.62 Hz, 1H, Ar-*H* C-2). RMN ^13^C (100 MHz, CD_3_OD): δ 151.1 (C-2), 98.28 (C-3), 151.4 (C-4), 124.6 (C-5), 123.08 (C-6), 134.9 (C-7), 126.2 (C-8), 148.2 (C-9), 117.32 (C-10), 50.03 (C-12), 42.08 (C-13), 39.9 (C-15), 38.46 (C-16).

BAQ, *N^1^-(7-chloroquinol-4-yl)-N^2^-{2-[(7-chloroquinol-4-il)amino]ethyl}ethane-1,2-diamine*, was synthesized according to the synthesis procedures described by Zhang and collaborators [40]. Briefly, a mixture of 4,7-dichloroquinoline (1.20 g, 6.00 mmol) and diethylenetriamine (0.22 mL, 2.00 mmol) was heated at 80°C for 6 h without stirring and then at 130°C for 3 h with stirring. The reaction mixture was cooled to room temperature and the resulting solid that formed was washed with an aqueous solution of 10% NaOH (30 mL) and recrystallized with MeOH/water. The solid was recuperated by filtration and dried under vacuum, which lead to a white solid. Chemical and spectral analyses revealed the following: Yield: 0.43 g, 50%. M.F: C_22_H_21_Cl_2_N_5_; M.M.: 426.34 g/mol. Elem. Anal., theoretical: C_22_H_21_Cl_2_N_5_, C, 61.98; H, 4.96; N, 16.43; experimental: C_22_H_21_Cl_2_N_5_.4H_2_O, C, 53.03; H, 4.91; N, 14.07. M.P.: 212–215°C; IR. (ν_max_ in cm^−1^): 3272 (Ar- NH); 3071 (CH quinoline ring); 2901 and 2843 (CH_2 as/s_); 1608, 1576 and 1531 (C = C quinoline ring); 1441 (CH_2_); 1328 (Ar-CN); 1136 (C-N); 865 and 798 (δ out-of-plane aromatic ring). ^1^H RMN (400 MHz, CD_3_OD): δ 2.97 (t, J = 6.22 Hz, 2H, CH_2_-13), 3.45 (t, *J* = 6.24 Hz, 2H, CH_2_-12), 6.45 (d, *J* = 5.69 Hz, 1H, Ar-*H* C-3), 7.20 (dd, *J* = 9.01 Hz, *J* = 2.14 Hz, 1H, Ar-*H* C-6), 7.67 (d, *J* = 2.12 Hz, 1H, Ar-*H* C-8), 7.87 (d, J = 8.97 Hz, Ar-*H* C-5), 8.24 (d, J = 5.61 Hz, 1H, Ar-*H* C-2). RMN ^13^C (100 MHz, MeOD): δ 150.9 (C-2), 98.3 (C-3), 151.2 (C-4), 124.6 (C-5), 122.6 (C-6), 134.9 (C-7), 126.2 (C-8), 148.14 (C-9), 117.3 (C-10), 46.8 (C-12), 41.8 (C-13).

### Continuous cultures of *P. falciparum*

*P. falciparum* blood parasites (W2 clone, CQ-resistant and mefloquine-sensitive) were maintained in continuous culture as previously described [41] with slight modifications [42]. The 3D7 strain, CQ- sensitive, originally received from the New York University Medical School, was also used. Briefly, the parasites were kept at 37°C in human erythrocytes (A^+^) with complete medium (RPMI 1640 supplemented with 10% human serum blood group A^+^, 2% glutamine and 7.5% NaHCO_3_) in Petri dishes in a candle jar or in a 25-cm culture flask in a defined atmosphere (with 3% O_2_, 5% CO_2_ and 91% N_2_). Human erythrocytes (A^+^) and serum blood group A+ were kindly donated by Center of Hemotherapy and Hemathology of Minas Gerais (HEMOMINAS). Immediately before the tests, ring stage-parasites were synchronized in sorbitol [43]. Synchronized parasites were stained with orange green in pre-dried coverslips [44] then immediately examined under a fluorescent microscope. The suspension was adjusted for parasitemia and hematocrit as described below for each test. The infected red blood cells were distributed in a 96-well microtiter plate (Corning, Santa Clara, CA, EUA) at 180 µL/well, in which 20 µL of the test drugs and controls were previously diluted at different concentrations (400-0.625 ng/mL) in 0.005% DMSO (v/v) (Sigma, USA).

### *In vitro* assays with *P. falciparum* infected erythrocytes

The effects of BAQ and MAQ on the W2 and 3D7 *P. falciparum* blood cultures were assayed through the incorporation of ^3^H hypoxanthine (PerkinElmer, Waltham, MA, EUA) [45] and with monoclonal antibodies to the parasite histidine and alanine-rich protein (HRPII) (MPFM ICLLAB-55A®, MPFG55P ICLLAB®, USA) (Noedl 2002). The [^3^H]-hypoxanthine assay was performed at 1% parasitemia and 1% hematocrit and the level of isotope incorporation was read in a beta-counter (PerkinElmer, Waltham, MA, EUA). The anti-HRPII test was performed at 0.05% parasitemia and 1.5% hematocrit and the HRPII quantification was read at 450 nm in a spectrophotometer (SpectraMax340PC^384^, Molecular Devices). The results of three of these drug activity assays were expressed as the mean of the half-maximal inhibitory dose (IC_50_) and compared with the drug-free controls. Curve-fitting was performed using Origin 8.0 software (OriginLab Corporation, Northampton, MA, USA) [46].

### Inhibition of hemozoin assay

The hemozoin inhibition assay was performed as described by Ncokazi et al., (2002) [47] with some modifications. Briefly, 10.1 µL of the aminoquinoline derivatives at different concentrations (40-0.62 mg/mL) and 101.2 µL of the hematin stock solution (1.68 mM in 0.1 M NaOH) were combined in triplicate in a flat-bottomed 96-well plate. The solutions were further mixed and 58.7 µL of acetate solution (12.9 M, pH 5.0) then added to each well. After incubation at 60°C, 80 µL of 30% (v/v) pyridine solution in 20 mM HEPES, pH 7.5, were added at room temperature. The solids formed were resuspended and allowed to settle for 15 min at room temperature. Then, 38 µL of the supernatant were transferred to a new plate and diluted to 250 µL with a 30% (v/v) pyridine solution (20 mM HEPES, pH 7.5). The color reaction was read in a spectophometer at 405 nm and the results were expressed as the concentration of free heme in each well, which was calculated from the standard absorbance curve of a known hemin solution. CQ was used as a positive control and the *cut off* was determined by the amount of free heme in the drug-free control. Each compound was evaluated in two different experiments.

### Antimalarial tests against *P. berghei* in mice

A suppressive parasite growth test was used with *P. berghei*, NK65 strain (originally received from the New York University Medical School) infected mice as described previously [48] with some modifications [49]. Briefly, adult Swiss outbred mice (20±2 g weigh) were inoculated with 1×10^5^ red blood cells infected with *P. berghei*, by intraperitoneal route. The infected mice were maintained together for at least 2 h and then divided randomly into groups of 3–5 animals per cage, which were subsequently treated with 25 and 50 mg/kg of each compound diluted in DMSO 3% (v/v) given daily by oral gavage for three consecutive days. Two control groups were used in parallel, one treated with CQ (20 mg/kg) and one with the vehicle. Blood smears from mouse tails were prepared on days 5, 8 and 10 post-infection and then methanol-fixed, stained with Giemsa and examined microscopically. The parasitemia was evaluated and the percent inhibition of parasite growth calculated in relation to the untreated control group (considered 100% growth) using the following equation: [(C - T)/C] ×100; where C is the parasitemia in the control group and T is the parasitemia in the treated group.

The use of laboratory animals was approved by the Ethics Committee for Animal Use of the Oswaldo Cruz Foundation - Fiocruz (CEUA L-0046/08).

### Cell cultures and cytotoxicity tests

The human hepatoma cell line (HepG2) was originally received from the New University of Lisbon, and the monkey kidney cell line (BGM) from the Federal University of Minas Gerais. These cells were cultured in 75 cm^2^ plates with RPMI 1640 medium supplemented with 10% heat-inactivated fetal calf serum and 40 mg/L gentamicine in a 5% CO_2_ atmosphere at 37°C. For the *in vitro* tests, a confluent cell monolayer was trypsinized, washed with culture medium, distributed in a flat-bottomed 96-well plate (5×10^3^ cells/well) and incubated for 18 h at 37°C to ensure cell adherence.

The cytotoxicity was evaluated with the MTT assay, 3-(4,5-Dimethylthiazol-2-yl)-2,5-diphenyltetrazolium bromide [50]. Briefly, the HepG2 and BGM cell lines were incubated with 20 µL of the compounds at different concentrations (1000-1 µg/mL) for 24 h in a 5% CO_2_ and air atmosphere at 37°C. For the MTT assay, which evaluates mitochondrial viability, 20 µL of MTT solution (5 mg/mL) were added and the plates were incubated for a further 3 h. After incubation, the supernatant was carefully removed from the wells, followed by the addition of 100 µL DMSO with thorough mixing. The optical density at 570 and 630 nm (background) was determined on an ELISA reader (SpectraMax340PC^384^, Molecular Devices).

The neutral red assay was used to evaluate cell viability by the accumulation of dye in the viable cell lysosome [51]. Briefly, the cells were incubated for 2 h with 100 µL of a neutral red solution (100 µg/mL). Then, the supernatants were discard and 200 µL of a formaldehyde (0.5% v/v) and CaCl_2_ (1%) solution were added, which was followed by another 5 min incubation. Finally, the supernatant was removed, then 100 µL of an alcohol-acid solution added to the sediment. The absorbance was read at 540 nm on an ELISA reader (SpectraMax340PC^384^, Molecular Devices). Cell viability was expressed as the percentage of control absorbance obtained in untreated cells after subtracting the absorbance of the appropriate background. Lastly, the minimum lethal dose for 50% of the cells (MLD_50_) was determined as previously described [52].

### Hemolysis assay

The test and control compounds were diluted in a 0.2% (v/v) DMSO solution and incubated (0–50 µg/mL) with a human erythrocytes suspension (1% hematocrit) at 37°C for 30 min in a shaking water bath. The mixtures were centrifuged at 1000 g for 10 min and the absorbance of the supernatants was measured at 540 nm in an ELISA reader (SpectraMax340PC^384^, Molecular Devices). The hemolytic rate was calculated in relation to the hemolysis of erythrocytes in 0.05% saponin, which was considered to be 100% [53].

### Docking energy calculations

The 3D optimized structure of dimeric hematin was taken from our previous studies [29]. The structures of CQ and its analogs were built using the Gaussian View software and optimized using Gaussian 03 [54]. The structural analysis of CQ analogs (BAQ and MAQ) was performed and all possible protonation forms in the biological medium were considered (Figure 2). The geometry optimizations and partial atomic charge distributions were performed using the B3LYP/3-21G level of theory. The calculations for the docking energies were performed with Molegro Virtual Docker (MVD) software [55] as described previously [25]. The algorithm used in the docking studies was the MolDock Score, which is an adaptation of the Differential Evolution (DE) algorithm [53]. The MolDock Score was used because it yields a higher docking accuracy than other state-of-the-art docking algorithms (MVD: 87%, Glide: 82%, Surflex: 75%, FlexX: 58%) [46].

The 3D structure of PfLDH complexed with NADH and the substrate oxamate used in the present work was obtained from the Protein Data Bank (PDB) file 1LDG [56], [57]. We performed flexible docking studies (considering the presence of water molecules in the crystal) with the protonated forms of BAQ and MAQ to compare these interactions with protonated chloroquine at the active site of PfLDH.

## Supporting Information

Scheme S1 Synthesis of MAQ and BAQ.
(TIF)
