# Supplementary figures and images for: Antimalarial Activity and Mechanisms of Action of Two Novel 4-Aminoquinolines against Chloroquine-Resistant Parasites
Source: PLoS One. 2012 May 23;7(5):e37259. doi: 10.1371/journal.pone.0037259 (PMC3359361; doi:10.1371/journal.pone.0037259)

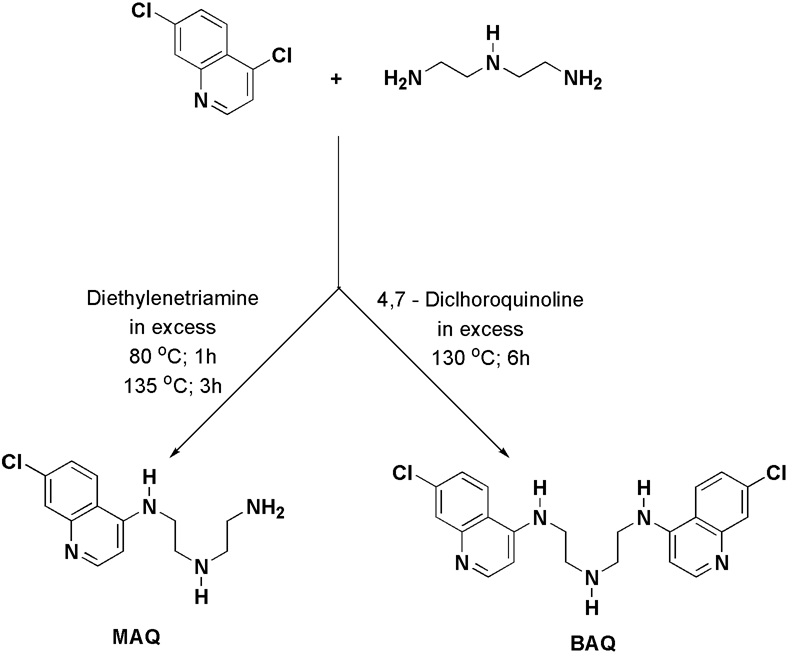

Supplement: Scheme S1 — Synthesis of MAQ and BAQ. (TIF) [file pone.0037259.s001.tif]
